# Presence and significance of telocytes in cholelithiasis and biliary dilatation in benign biliary disorders

**DOI:** 10.1038/s41598-024-65776-w

**Published:** 2024-06-28

**Authors:** Gongqing Ren, Ruizi Zhong, Gang Zou, Hongling Du, Yue Zhang

**Affiliations:** 1 The Second Clinical Medical College, Jinan University, Shenzhen, China; 2 Department of Hepatobiliary Pancreatic Surgery, Shenzhen People’s Hospital, No.1017 Dongmen North Road, Shenzhen, 518020 Guangdong Province China; 3 Department of Burns and Plastic Surgery, Shenzhen People’s Hospital, Shenzhen, China; 4 Department of Thyroid and Breast Surgery, The Second Affiliated Hospital of Guizhou Medical University, Kaili, China

**Keywords:** Telocytes, Benign biliary disorders, Gallstone disease, Biliary dilation syndrome, Pathophysiology, Cell biology, Molecular biology, Pathogenesis

## Abstract

Telocytes are closely associated with the regulation of tissue smooth muscle dynamics in digestive system disorders. They are widely distributed in the biliary system and exert their influence on biliary motility through mechanisms such as the regulation of CCK and their electrophysiological effects on smooth muscle cells. To investigate the relationship between telocytes and benign biliary diseases,such as gallbladder stone disease and biliary dilation syndrome, we conducted histopathological analysis on tissues affected by these conditions. Additionally, we performed immunohistochemistry and immunofluorescence double staining experiments for telocytes. The results indicate that the quantity of telocytes in the gallbladder and bile duct is significantly lower in pathological conditions compared to the control group. This reveals a close association between the decrease in telocyte quantity and impaired gallbladder motility and biliary fibrosis. Furthermore, further investigations have shown a correlation between telocytes in cholesterol gallstones and cholecystokinin-A receptor (CCK-AR), suggesting that elevated cholesterol levels may impair telocytes, leading to a reduction in the quantity of CCK-AR and ultimately resulting in impaired gallbladder motility.Therefore, we hypothesize that telocytes may play a crucial role in maintaining biliary homeostasis, and their deficiency may be associated with the development of benign biliary diseases, including gallstone disease and biliary dilation.

## Introduction

Benign biliary disorders encompass a spectrum of congenital and acquired conditions affecting the biliary system. Some of these conditions may involve the intrahepatic or extrahepatic bile ducts, often presenting as either acute or chronic ailments, with the potential to progress into malignancies^1^. Common benign biliary disorders include gallstone disease, choledocholithiasis, and biliary dilation syndrome. The exact etiology of gallstone disease remains unclear. Acute cholecystitis, often precipitated by gallstones, exhibits a high global incidence and imposes a significant economic burden. The prevalence of gallstone disease ranges from 10 to 20% in Europe and the United States^2^. In China, the prevalence of gallstone disease has also reached 10%, with a growing trend in recent years. Failure to promptly diagnose and treat gallstone disease can lead to serious consequences, such as cholangitis, pancreatitis, peritonitis, and even gallbladder cancer, placing a considerable financial burden on patients. In the United States, the direct and indirect economic expenditures for the treatment of gallstone disease exceed 6 billion dollars annually. In China, the average medical cost for gallstone patients requiring cholecystectomy is approximately 12,000 RMB, and the healthcare economic impact of conservative treatment and the management of associated complications in gallstone patients is substantial. While biliary dilation syndrome has a lower incidence compared to gallstone disease and is less common in adults than in children^3,4^, it can still lead to complications such as cholelithiasis, cholangitis, pancreatitis, and bile duct perforation. Among these, the most severe complication is biliary malignancy, with approximately 62.3% of patients with concurrent biliary malignancies having gallbladder cancer and 32.1% having bile duct cancer^5^. Therefore, biliary dilation syndrome is also a notable benign biliary disorder. Despite the unclear etiology of these diseases, it has been observed in the study of their pathogenesis that smooth muscle dysregulation in gallbladder and biliary tissues plays a significant role.

Telocytes (TCs) are a distinct type of interstitial cells with morphological features resembling those of Interstitial Cells of Cajal (ICC), which are commonly found in the gastrointestinal tract. Professor Popescu identified similar cells with ICC-like morphology in various organs, including the pancreas^6^, heart^7^ and biliary system^8^. To avoid confusion with ICC, Professor Popescu officially coined the term ‘telocytes’ in 2010^9^. Research has shown that telocytes are associated with the contractile function of the gallbladder and extrahepatic bile ducts. A study^10^ demonstrated that guinea pig gallbladder telocytes express cholecystokinin-A receptor (CCK-AR) and confirmed that cholecystokinin (CCK) induces gallbladder contraction mediated by CCK-AR on telocytes. The extrahepatic bile ducts are also regulated by CCK, and the density of CCK-AR increases gradually in the lower segment of the common bile duct. CCK can directly bind to specific CCK-AR on smooth muscle cells, leading to the activation of the phospholipase C-coupled signaling pathway, which hydrolyzes phosphatidylinositol bisphosphate (PIP2) into inositol trisphosphate (IP3). IP3 then binds to IP3 receptor-gated calcium channels on the endoplasmic reticulum, triggering calcium release, increasing intracellular calcium concentration, and activating calcium-dependent contractile proteins, ultimately causing smooth muscle contraction. Moreover, telocytes are electrically coupled to smooth muscle cells (SMCs), potentially contributing to the generation and propagation of spontaneous rhythmicity in the gallbladder wall, regulating smooth muscle movement^11^. Fu Siyi et al.^12^ also found a significant reduction in liver telocytes by 27–60% in human liver fibrosis. This reduction may lead to alterations in the extracellular matrix, loss of control over fibroblasts/myofibroblasts’ activity, and subsequently promote fibrogenesis. Additionally, telocytes have various functions, including properties of mesenchymal stem cells, providing mechanical support during intestinal motility, participating in tissue homeostasis maintenance and regeneration, and influencing organ development^13,14^.

Due to the increased focus on the regulation of tissue smooth muscle dynamics in recent years in the study of gastrointestinal diseases, telocytes have become a hot topic in the field of investigating tissue smooth muscle dynamics^15^. To distinguish telocytes in the digestive system, the gene expression product of C-kit/CD117 is often used as a characteristic immunomarker for telocytes^16^. However, since mast cells also express C-kit to varying degrees, we use mast cell-specific immunomarker tryptase^17^ in conjunction with immunofluorescence staining techniques to simultaneously stain for C-kit and tryptase, allowing for precise identification of telocytes in the tissue. Particularly in the study of the role of telocytes in the bile ducts, to better understand the pathological changes of telocytes in biliary dilation syndrome, we also employ double immunofluorescence staining with the key apoptosis effector protein Caspase-3 to assess the apoptosis status of telocytes in the bile ducts. By investigating the expression patterns and characteristics of telocytes in gallstone disease and biliary dilation syndrome, we aim to summarize the pathophysiological roles of telocytes in benign biliary disorders.

## Materials and methods

### Tissue preparation

This study has received approval from the Medical Ethics Committee of Shenzhen People’s Hospital. All subjects have been duly informed of the research objectives and have provided signed informed consent forms. Gallbladder tissue specimens were collected between 2014 and 2020, sourced from patients undergoing cholecystectomy at Shenzhen People’s Hospital. This cohort includes 15 non-randomly selected patients with gallbladder polyps. Additionally, Fourier-transform infrared spectroscopy was employed to analyze the composition of gallstones. Fifteen cases each of non-randomly selected patients with cholesterol-type and pigment-type stones were chosen. A total of 45 gallbladder tissue samples were utilized, excluding patients with concurrent common bile duct stones or acute cholecystitis, as well as those with significant underlying organ pathologies in the heart, cerebrovascular system, liver, kidneys, lungs, etc. Patients with a history of previous biliary system surgery were also excluded.

Bile duct tissue specimens were collected between January 2017 and August 2021, from cases diagnosed with bile duct dilation at Shenzhen People’s Hospital. The experimental group comprised 26 patients who consented to participate in the study and underwent surgical resection of the dilated bile ducts. The control group included 6 patients who underwent surgical resection for reasons unrelated to biliary tract diseases, providing normal bile duct tissue. Among the 32 bile duct tissue samples, those resulting from biliary ductal dilation caused by bile duct stones or malignant tumors were excluded. Additionally, samples from patients with Caroli’s disease and those discovered to have bile duct damage after selection into the control group were also excluded.

The freshly resected gallbladder and bile duct tissues obtained from the surgery were sent to the pathology department for standard processing. The specimens of bile duct dilation were sectioned horizontally and vertically to expose the dilated bile duct tissue, while the specimens of normal bile duct were sectioned horizontally and vertically to expose the bile duct tissue. The specimens were thoroughly rinsed with PBS. Then, the specimens were placed in a universal tissue fixative for fixation. After dehydration, they were embedded in metal embedding molds. Before embedding, the desired section was oriented downwards to facilitate obtaining the desired area during sectioning. Molten paraffin was then poured into the molds using a pathology paraffin embedding machine. After cooling and solidification, the paraffin blocks were archived for future use.

This study was approved by the Ethics Committee of Shenzhen People’s Hospital, (approval number: LL-KY-2021559; LL-KT-2019263), Patients were consented by an informed consent process that was reviewed by the Ethics Committee of Shenzhen People’s Hospital and certify that the study was performed in accordance with the ethical standards as laid down in the 1964 Declaration of Helsinki.

### Fourier infrared spectroscopy analysis of gallbladder stones

Grind the collected gallstones into powder, dry and weigh 1–2 mg. Mix the powder with 200 mg of pure KBr and grind it to a particle size less than 2 µm. Use an infrared press machine to compress the powder into transparent thin films under a pressure of (5 − 10) × 107 Pa. Prepare a blank KBr film using the same method as described above. Open the Fourier transform infrared spectrometer and place the prepared KBr blank film on the sample holder in the sample compartment. Collect the reference background and then analyze the gallstone specimens one by one as required to obtain specific composition data of the gallstones. Use the Fourier transform infrared spectrometer to measure and analyze two different parts of the same gallstone, obtaining measurement peaks. Compare the characteristic peaks of cholesterol and bilirubin and use software to analyze the percentage of cholesterol and bilirubin in the gallstones. Select cholesterol-type and bilirubin-type gallstones that meet the criteria based on the experimental design.Histopathological analysisAfter embedding and fixation of the prepared wax blocks, consecutive sections were obtained using a tissue microtome and attached to appropriate positions on glass slides. The slides were then placed in a 37 °C incubator overnight. The selected paraffin-embedded sections underwent deparaffinization and were stained with hematoxylin and eosin for general histopathological analysis.

### Immunohistochemistry

After deparaffinization, paraffin-embedded tissue slices underwent antigen retrieval using the Heat-Induced Epitope Retrieval (HIER) method. Citrate antigen retrieval buffer, prepared by dissolving citrate antigen retrieval powder in distilled water to achieve a pH of 6.0, was used to immerse the tissue slices. These were then heated in a high-pressure steam cooker, maintaining the steam release for 2 min before allowing the slices to cool naturally to room temperature. To minimize nonspecific binding and improve antibody penetration, slices were pre-incubated with 5% normal goat serum for 20 min. Regions for antibody application were marked using an immunohistochemistry pen, and the primary antibody, rabbit anti-human C-kit polyclonal antibody (1:200, Dako, A450229-2), was applied. The slides were refrigerated at 4 °C overnight, followed by a 30-min room temperature incubation the next day. After three 5-min washes in PBS with gentle agitation, the slices were slightly air-dried and treated with a goat anti-mouse secondary antibody labeled with HRP. After incubating for 1 h at room temperature, the slices were washed three times in PBS, each for 5 min. DAB chromogen was then applied to designated areas, and development was monitored under a microscope until positive staining appeared as brown-yellow. To conclude the staining process, the slices were rinsed with distilled water, halting the reaction. Subsequent steps mirrored those of the Hematoxylin and Eosin (HE) staining protocol, involving repeated deparaffinization and dehydration. For negative controls, PBS replaced the primary antibody, following the outlined procedure. The effectiveness of the antibody staining was then evaluated microscopically, with relevant images captured for analysis.

### Immunofluorescent double staining

Deparaffinization of the paraffin-embedded slices was conducted as previously outlined, followed by Heat-Induced Epitope Retrieval (HIER) for tissue antigen extraction. The primary antibodies, prepared in PBS, were then applied specifically: for the gallbladder, a combination of rabbit polyclonal anti-human c-kit antibody (1:100, Dako, A450229-2) and rabbit anti-human mast cell chymase Tryptase antibody (1:100, Abcam, ab196772), and for the bile duct, rabbit polyclonal anti-human C-kit antibody (1:200, Dako, A450229-2) with mouse anti-human Caspase-3 monoclonal antibody (1:100, Invitrogen, 74T2). After overnight incubation at 4 °C, the slices were normalized to room temperature, rinsed thrice with PBS, and treated with Alexa Fluor 555-labeled goat anti-mouse secondary antibodies (1:500, Invitrogen, A-21422)and Alexa Fluor 488-labeled goat anti-rabbit secondary antibodies (1:500, Invitrogen, A-11008). Post one hour of incubation in dim light, they were washed with PBS. DAPI staining was then applied for three minutes in darkness, followed by three five-minute PBS washes on a destaining rocker. Conclusively, an anti-fluorescence quenching agent was used before the slices were sealed, imaged, and analyzed with a fluorescent microscope.

### Statistical analysis

We conducted data analysis on telocytes research in the gallbladder and bile duct using the SPSS software. For gallbladder data, under the assumption of homogeneity of variances, we performed one-way analysis of variance (One Way ANOVA) along with the LSD test for intergroup comparisons. The relationship between telocyte quantity and CCK-AR expression was assessed using Pearson correlation analysis. Regarding the bile duct, when data met the homogeneity of variances assumption, we conducted independent samples t-tests for comparison. In cases of heteroscedasticity, we opted for a *corrected t-test (t’ test)*. Statistically significant differences were considered when the *P* < *0.05*.

## Results

### Characteristic peaks of different components of gallbladder stones

**Figure 1:**
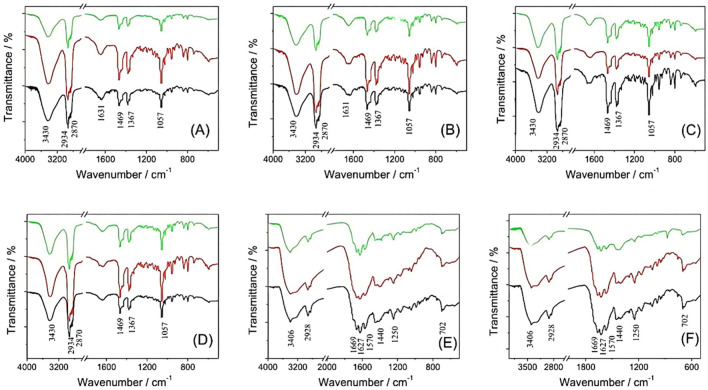
Representative infrared spectra of different types of gallstones Cholesterol type (**A**–**D**); Bile pigment type (**E**, **F**).

Upon observation, resected gallbladder stones can be primarily categorized into two color-based classifications: yellow and black. Yellow gallbladder stones typically exhibit a polyhedral or elliptical shape with a smooth surface, primarily composed of cholesterol. In contrast, the majority of black gallbladder stones present a coal-like appearance, are characterized by a hard texture, and possess a rough surface, primarily consisting of bilirubin. Through Fourier-transform infrared spectroscopy analysis, distinct characteristic peaks were identified for cholesterol stones, including v = 1057, 1367, 1469, 2870, 2934, and 3430 cm^−1^. Conversely, bilirubin stones exhibited the following characteristic peaks: v = 702, 1250, 1440, 1570, 1627, 1669, 2928, and 3406 cm^−1^ (Fig. 1).

### Histopathological analysis of the gallbladder and bile ducts

**Figure 2:**
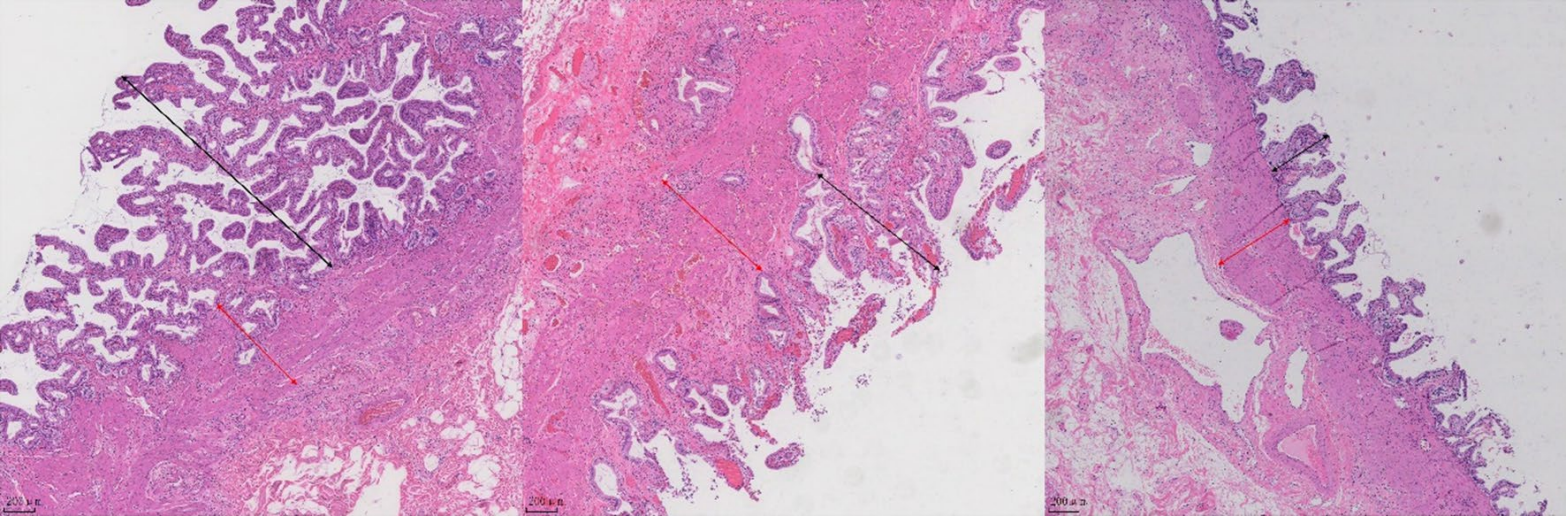
Comparison of gallbladder wall thickness among the groups Thickness of the submucosal layer (black bidirectional arrows); thickness of the muscular layer (red bidirectional arrows). (**A**) Cholesterol gallstone group; (**B**) Bilirubin gallstone group; (**C**) Gallbladder polyp group.

Upon microscopic examination of the H&E stained sections from all groups, in the gallbladder, a comparison with the gallbladder polyp group revealed that experimental group A exhibited noticeable infiltration of inflammatory cells in the gallbladder mucosal epithelium, accompanied by signs of hyperplasia and thickening. The thickness of the muscular layer was significantly reduced and displayed disordered arrangement. In contrast, in experimental group B, there were no prominent signs of epithelial thickening in the gallbladder mucosa, but a reduction in muscular layer thickness and disorganized alignment were observed (Fig. 2). It’s important to note that no significant fibrotic or tumorous alterations were observed in any of the groups.

**Figure 3:**
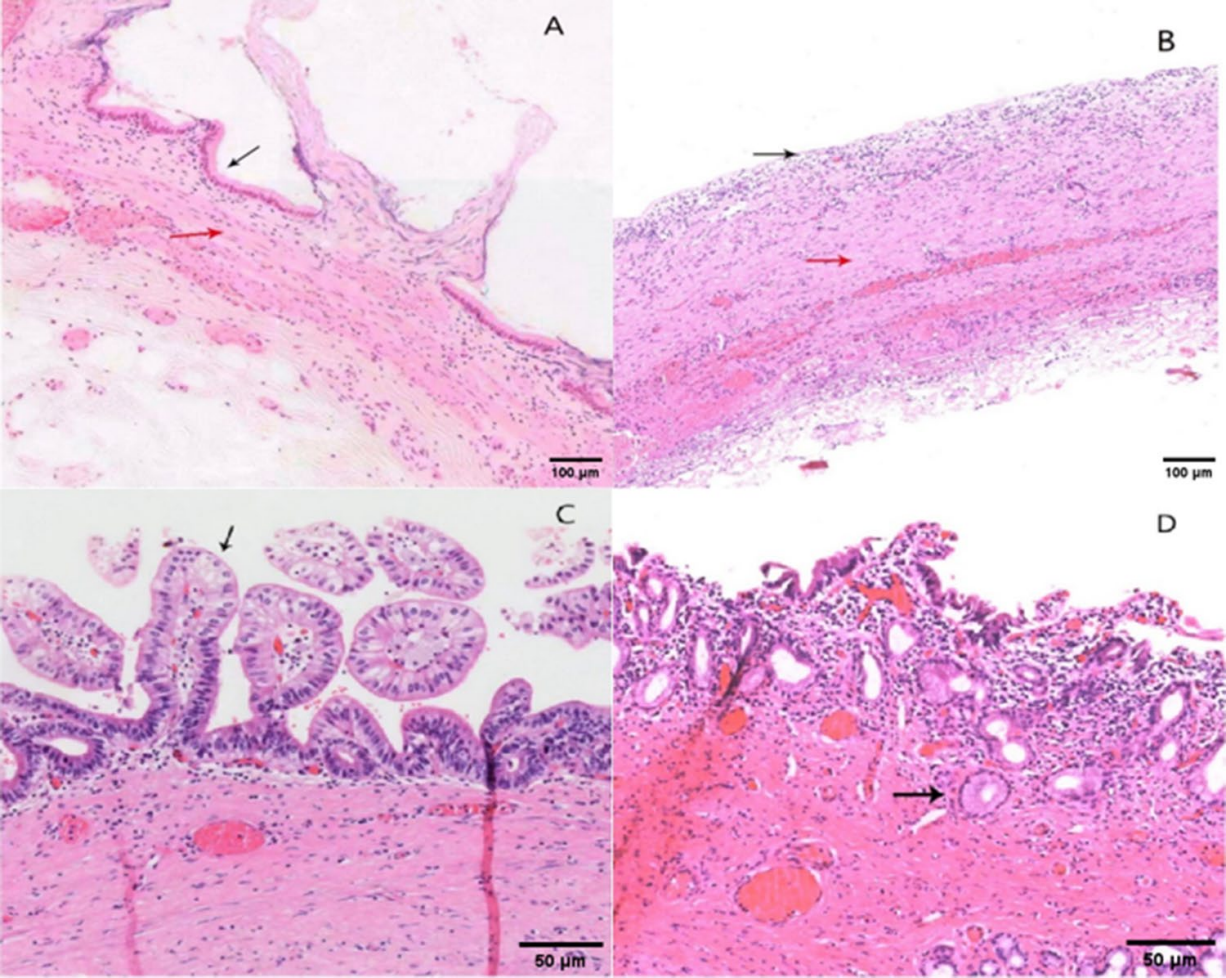
Comparison of Normal Bile Ducts and Dilated Bile Ducts in HE Staining Control Group (**A**) Normal bile duct epithelium is discernible (indicated by the black arrow), along with muscular tissue (indicated by the red arrow); (× 40) Experimental Group (**B**) Epithelial cells have detached (indicated by the black arrow), submucosa exhibits a substantial aggregation of inflammatory cells, accompanied by visible fibrous tissue (indicated by the red arrow); (× 40) Experimental Group (**C**) Intestinal epithelial metaplasia is observable (indicated by the black arrow); (**D**) Pyloric gland hyperplasia is evident (indicated by the black arrow); (× 100).

In the bile ducts of the normal control group, as compared to the control group, observations revealed that the experimental group exhibited mucosal epithelial sloughing and loss. Multiple circular areas of inflammatory cell infiltration were present in the submucosa, often accompanied by varying degrees of reduced muscular tissue. Additionally, there was a significant presence of loose filamentous fibrous tissue (Fig. 3B). Furthermore, intestinal metaplasia (Fig. 3C) and pyloric glandular metaplasia (Fig. 3D) were also observed. The outer membrane displayed vascular proliferation with local bleeding, and there was evidence of adhesions between the cyst wall and the surrounding structural tissues. The statistical data showed that among the 26 patients in the experimental group, the proportions of different pathological changes were as follows (Table 1): 92.3% of patients exhibited damage, sloughing, or loss of the bile duct mucosal epithelium.92.3% of patients displayed varying degrees of inflammatory infiltration, with the primary inflammatory cell types including lymphocytes, neutrophils, and plasma cells. Approximately 65.5% of patients had scattered muscular tissue loss or continuous or intermittent band-like distribution of muscular tissue. About 92.3% of patients exhibited varying degrees of fibrous tissue proliferation, with fibrosis often limited to two-thirds of the bile duct wall. Additionally, 38.4% of the bile ducts showed varying degrees of glandular proliferation, including 7 cases of pyloric glandular metaplasia (2 cases of cystic expansion and 5 cases of spindle-shaped expansion) and 3 cases of intestinal epithelial metaplasia (2 cases of cystic expansion and 1 case of spindle-shaped expansion) (Fig. 3A).

**Table 1:** The pathological changes in the bile ducts of patients with biliary dilation.

Rating/percentage	Extent of epithelial detachment	Extent of inflammatory infiltration	Smooth muscle fiber distribution	Extent of fibrosis
0	2 (7.7%)	2 (7.7)	10 (38.5%)	3 (11.5%)
1	4 (15.4%)	12 (46.1%)	7 (26.9%)	9 (34.6%)
2	6 (23.1%)	6 (23.1%)	6 (23.1%)	10 (38.5%)
3	14 (53.8%)	6 (23.1%)	3 (11.5%)	4 (15.4%)

### Immunohistochemistry of the gallbladder and bile ducts

**Figure 4:**
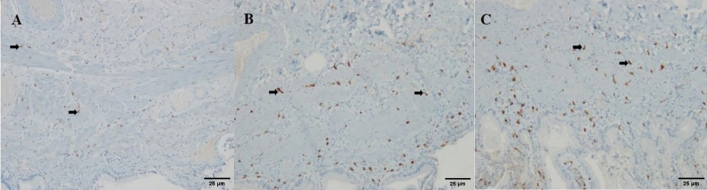
C-kit immunohistochemical results in the gallbladder among different groups, with brown cells (indicated by arrows) representing C-kit positive cells. (**A**) Cholesterol gallstone group; (**B**) Bilirubin gallstone group; (**C**) Gallbladder polyp group (Magnification: 10 × 20).

**Figure 5:**
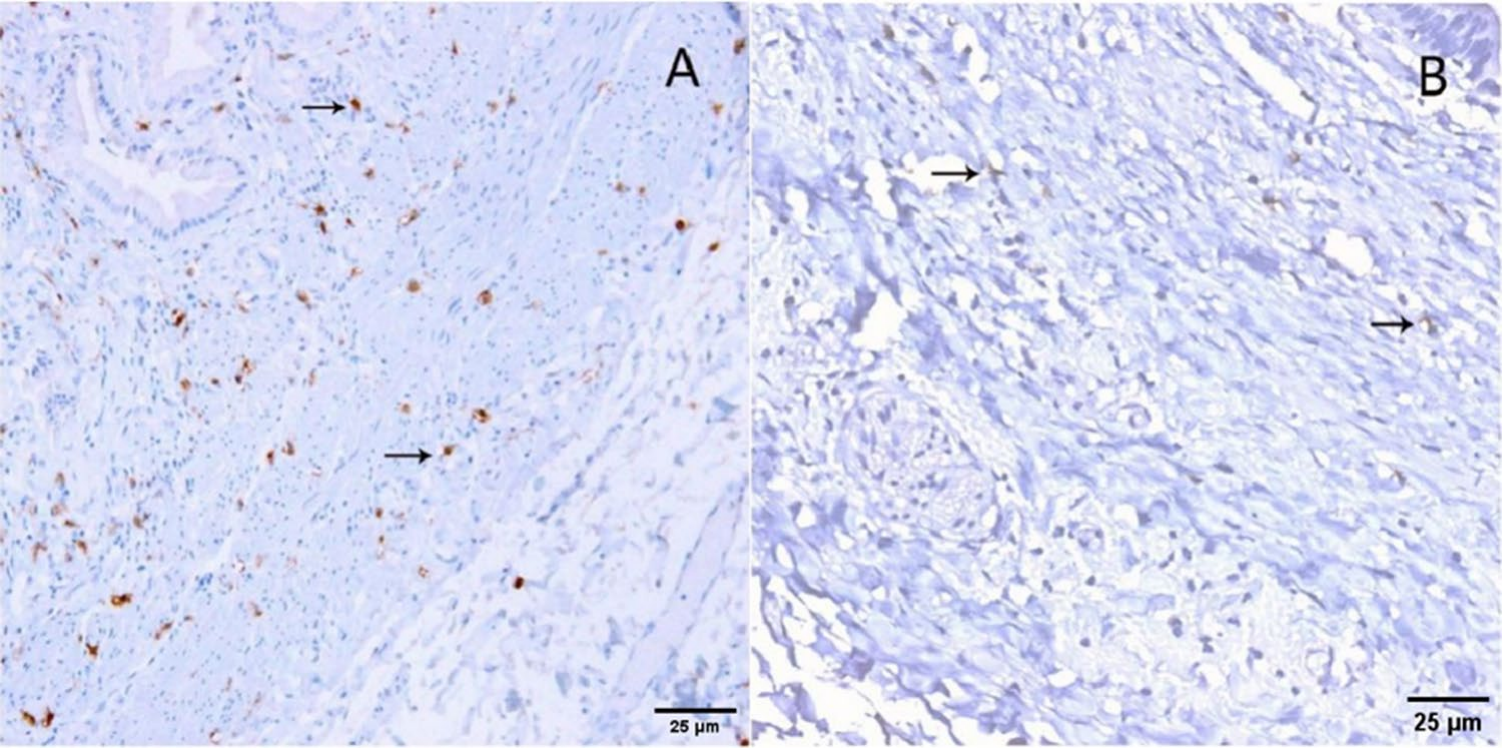
Comparison of C-kit Immunohistochemistry between Normal Bile Ducts and Ductal Dilatation in the Biliary Tract. (**A**) Control Group; (**B**) Experimental Group(Magnification: 10 × 20).

Using C-kit as the primary antibody for immunohistochemical staining, we observed the presence of C-kit positive cells distributed throughout various tissue layers. In the cholesterol gallstone group, the gallbladder muscular layer appeared thinner, and in comparison to the other two groups, the distribution of C-kit positive cells was relatively sparse. In the bilirubin gallstone group, C-kit positive cells exhibited deep staining, resembling their distribution pattern in the gallbladder polyp group (Fig. 4). In the control group of bile ducts, C-kit positive cells were predominantly densely concentrated within the bile duct muscular layer. However, in the experimental group, the number of C-kit positive cells was reduced, and their distribution was relatively more scattered (Fig. 5).

### Immunofluorescence double staining on the gallbladder

In the immunofluorescent double-staining experiment of the gallbladder, the control group exhibited a predominant distribution of telocytes in the interstitial spaces and around the muscle layers. These cells typically exhibited a spindle-shaped morphology with elongated processes along the longitudinal axis and were closely adjacent to the muscle cells within the muscular layer, displaying a parallel arrangement. In contrast, labeled mast cells displayed smaller, round-shaped cell bodies with centrally located cell nuclei. Mast cells were primarily distributed in the mucosal lamina propria but were also sparsely distributed in various layers of the tissue. Based on preliminary observations of cell morphology and distribution, we were able to distinguish telocytes from mast cells.

**Figure 6:**
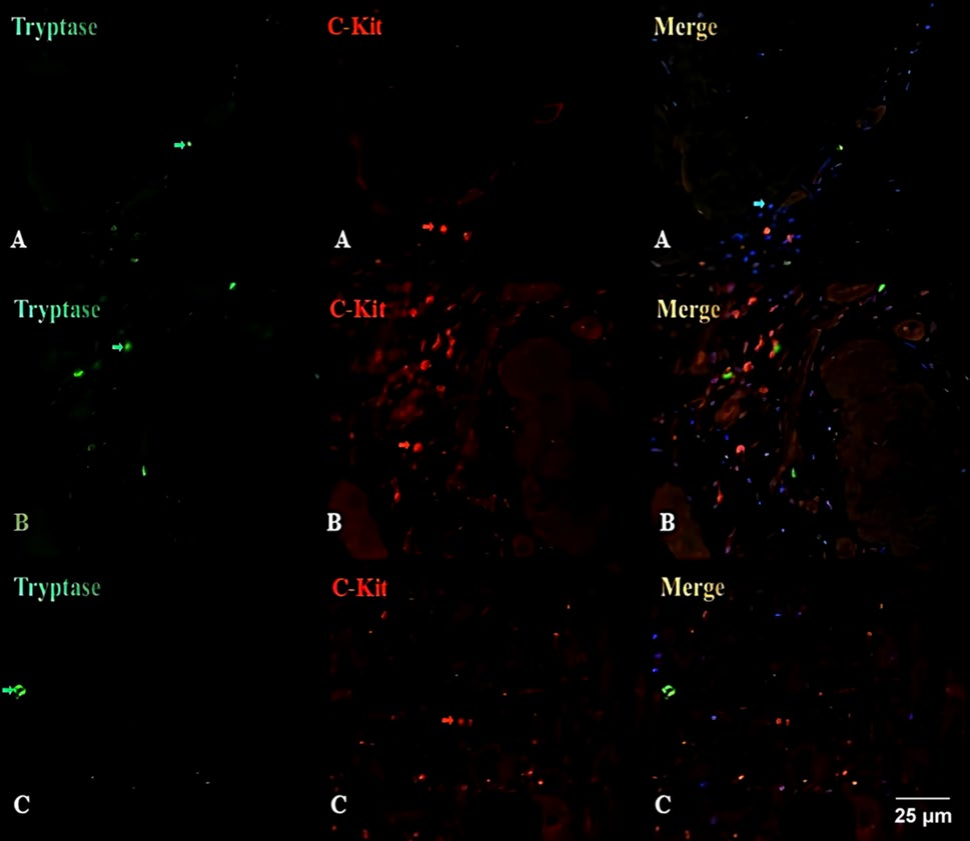
Immunofluorescent staining was conducted on gallbladder tissues in all groups using C-kit (red arrows) and Tryptase (green arrows) markers, with cell nuclei counterstained using DAPI (blue arrows). It was observed that the number of C-kit-positive and Tryptase-negative telocytes was notably sparse in the cholesterol gallstone group. In contrast, both the gallbladder polyp group and the bilirubin gallstone group exhibited a higher count of C-kit-positive and Tryptase-negative telocytes. (**A**) Cholesterol Gallstone Group; (**B**) Bilirubin Gallstone Group; (**C**) Gallbladder Polyp Group (10 × 40 magnification).

C-kit is a characteristic marker for telocytes, but it can also label some mast cells. Using corresponding fluorescent secondary antibodies, C-kit positive cells exhibit red fluorescence under a fluorescence microscope, while Tryptase antibody positive cells, representing mast cells, exhibit green fluorescence. CCK-AR positive cells, which represent gallbladder contraction function, also exhibit green fluorescence. If C-kit is co-labeled with Tryptase or CCK-AR, the fluorescence will combine to form orange. In the C-kit and Tryptase immunofluorescence assay, after capturing and analyzing images using a fluorescence microscope, only red fluorescence indicates telocytes (C-kit positive and Tryptase negative, which are the desired positive cells). In the C-kit and CCK-AR immunofluorescence assay, yellow fluorescence indicates co-expression of CCK-AR on C-kit cells (C-kit positive and CCK-AR negative). Under a fluorescence microscope at a magnification of 10 × 40, five positive fields were randomly selected for each slice using a blind method. Image-pro plus software was used to analyze the different immunofluorescence expressions and calculate and analyze the quantities of telocytes and CCK-AR positive cells. The results indicated a significant reduction in the distribution of telocytes in the cholesterol gallstone group compared to the control group, with a statistically significant difference (*LSD-t* = *19.24, P* = 0.000). In contrast, there was no statistically significant difference in the number of telocytes between the bilirubin gallstone group and the control group (*LSD-t* = *1.51, P* = 0.138). Furthermore, the comparative results in the experimental groups also indicated a statistically significant difference, with a lower quantity of telocytes in the cholesterol gallstone group compared to the bilirubin group (*LSD-t* = *17.73, P* = 0.000) (Table 2 and Fig. 6).

**Table 2:** Comparison of the number of gallbladder trophoblasts in three groups of patients.

Group	Number of examples	Number of telocytes
Experimental group A	15	11.49 ± 2.48
Experimental group B	15	43.09 ± 6.37
Control group	15	45.79 ± 4.98
Overall comparison: *F, P*		288.96, 0.000
Multiple comparison: *LSD-t*, *P*	Experimental group A versus experimental group B	*LSD-t* = 17.73* P* = 0.000
	Experimental group A versus control group	*LSD-t* = 19.24* P* = 0.000
	Experimental group B versus control group	*LSD-t* = 1.51* P* = 0.138

Experimental group A: cholesterol gallstone group; experimental group B: bilirubin gallstone group; control group: gallbladder polyp group.

**Figure 7:**
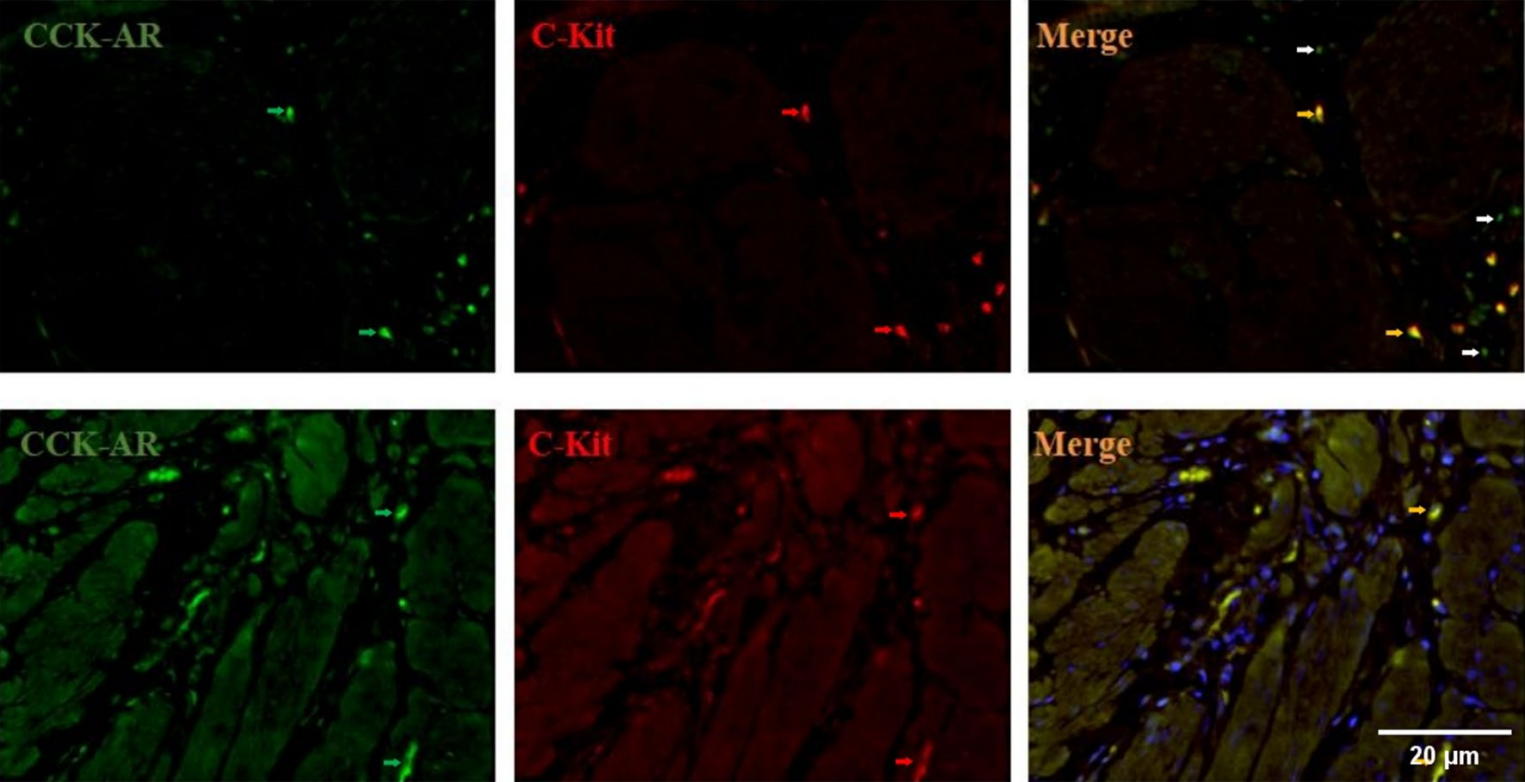
In the immunofluorescent staining of CCK-AR and C-kit, co-localization of C-kit and CCK-AR was observed in human gallbladder tissue. Most C-kit-positive cells exhibited the expression of CCK-AR, demonstrating morphological characteristics consistent with telocytes. However, CCK-AR expression was not universal among C-kit-positive cells (10 × 40 magnification).

We employed a dual immunofluorescence method to compare the quantity of telocytes and CCK-AR between different groups. Pearson correlation analysis was applied to examine the relationship between the quantity of telocytes and CCK-AR. It was noted that within the gallbladder muscle layer, there were double-positive cells for CCK-AR and C-kit. Analysis of the morphological features and distribution of C-kit-positive cells revealed that these cells exhibited characteristic morphology akin to that of telocytes. This suggests the presence of co-expression regions for CCK-AR on telocytes. Furthermore, aside from co-localized expression with C-kit, CCK-AR was also observed to be expressed individually in certain cells (Fig. 7).

**Figure 8:**
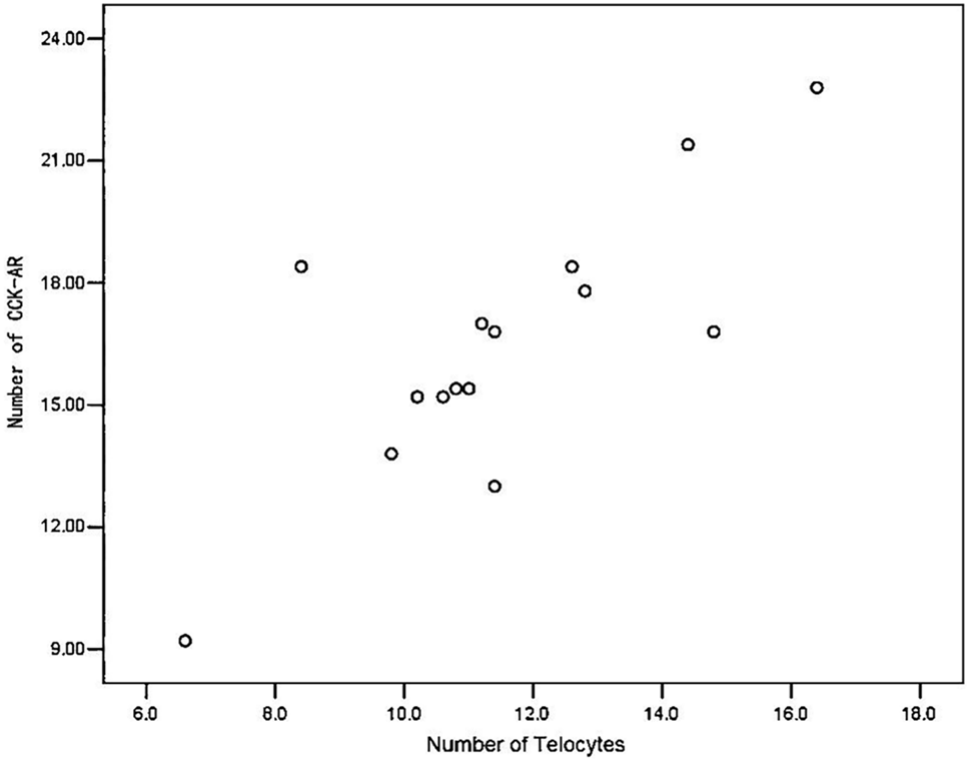
Analysis of the correlation between the number of telocytes in the human gallbladder and the number of CCK-AR.

Through the presentation of scatter plots, we observed a positive correlation trend between the number of CCK-AR cells and telocytes within the gallbladder in the cholesterol gallstone group. This trend manifested as a linear relationship (Fig. 8). Further Pearson correlation analysis results indicated a significant positive correlation (r = 0.776, *P* = 0.001) between the number of telocytes and CCK-AR cells in the gallbladder of cholesterol gallstone patients, signifying a notable association between telocytes and CCK-AR in the gallbladder of cholesterol gallstone patients.

### Immunofluorescence double staining on bile ducts

**Figure 9:**
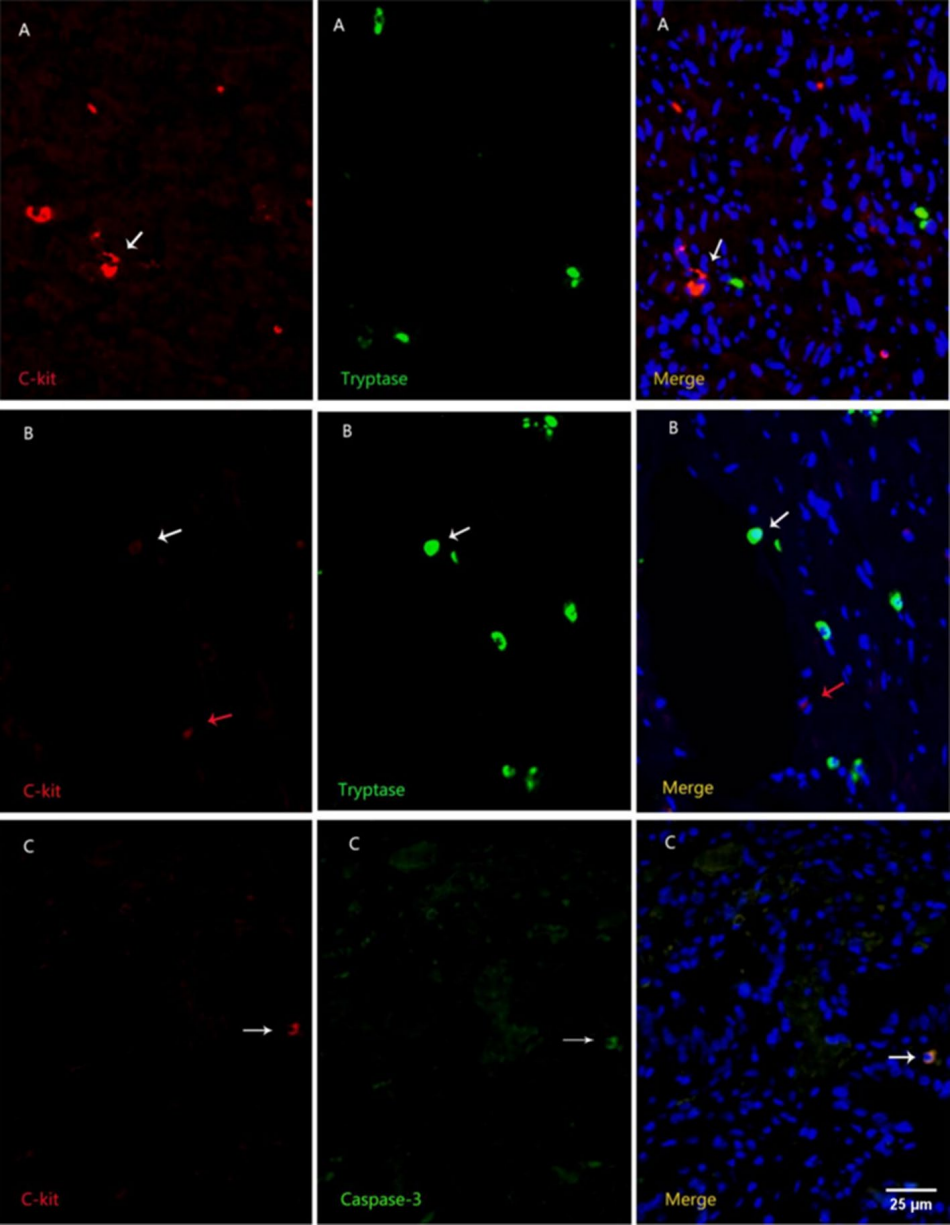
Comparison of C-kit with Tryptase and Caspase-3 dual immunofluorescence staining (× 200). Control group (**A**) Telocytes (white arrows). Experimental group (**B**) Telocytes (red arrows), enlarged mast cells positive for C-kit and Tryptase (white arrows). Experimental group (**C**) Cells doubly positive for C-kit and Caspase-3 (white arrows).

By employing dual immunofluorescence staining for C-kit and Tryptase (Fig. 9), we observed telocytes that were C-kit positive and Tryptase negative. These telocytes exhibited a red immunofluorescence signal and displayed diverse morphologies, including circular, spindle-shaped, and irregular forms. They were predominantly situated within the interstitial spaces and around the muscle layers. Simultaneously, mast cells, which were C-kit negative and Tryptase positive, displayed green immunofluorescence and typically exhibited a rounded shape, mainly distributed within the mucosal submucosa. A smaller quantity of these mast cells was found scattered throughout various tissue layers. Moreover, some cells demonstrated both C-kit and Tryptase positivity. Following DAPI counterstaining, the cell nuclei appeared blue.Additionally, in the context of dual immunofluorescence staining for C-kit and Caspase-3 (Fig. 9C), cells exhibiting dual positivity for C-kit and Caspase-3 emitted an orange hue, signifying the expression of Caspase-3 by telocytes.

The fluorescence staining of C-kit and mast cells in the bile duct tissue is the same as in the gallbladder tissue. In addition, Caspase-3 antibody positive cells, which serve as markers for apoptosis promotion, exhibit green fluorescence. If C-kit is co-labeled with Caspase-3, the fluorescence will combine to form orange. In the C-kit and Tryptase immunofluorescence assay, after capturing and analyzing images using a fluorescence microscope, only red fluorescence indicates telocytes (C-kit positive and Tryptase negative, which are the desired positive cells). In the C-kit and Caspase-3 immunofluorescence assay, orange fluorescence indicates co-expression of C-kit and Caspase-3 (C-kit positive and Caspase-3 positive). Under a fluorescence microscope at a magnification of 10 × 20, five non-overlapping fields were randomly selected for each slice using a blind method. Image-pro plus software was used to analyze the different immunofluorescence expressions and calculate and analyze the quantities of C-kit positive cells and C-kit + Caspase-3 co-expression cells.The statistical methodology remains consistent with previous approaches.

**Figure 10:**
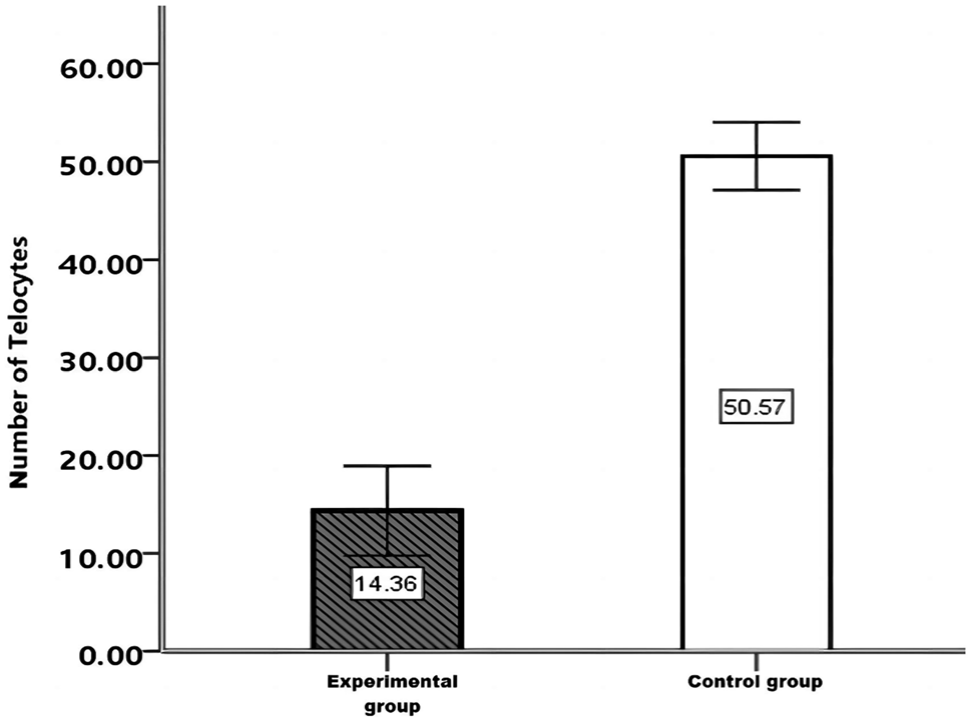
Comparison of telocyte counts between the experimental group and the control group in the biliary tract.

The comparison of telocyte quantities between the two groups through immunofluorescence dual staining (Fig. 10), revealed a significantly lower number of telocytes in the experimental group (14.6 ± 11.3) compared to the control group (50.6 ± 3.3). Levene’s test demonstrated a *P*-value of 0.005, indicating statistical significance (*t’* =  − 13.94, *P* = 0.000). Finally, the percentages of double-positive cells in the experimental and control groups were (8.9 ± 8.6%) and (3.0 ± 1.1%), respectively. Levene’s test showed a *P*-value of 0.006, confirming a statistically significant difference (*t’* = 3.39, *P* = 0.002).

## Discussion

Benign biliary tract disorders encompass a variety of conditions, while they may exhibit clinical and laboratory similarities, they are distinguished by significant disparities in etiology, treatment modalities, and prognosis at present^1^. To explore potential shared pathological mechanisms within these conditions, we have selected two prevalent benign biliary tract fundamental disorders, namely cholelithiasis and biliary dilatation. Our investigation delves into the expression of telocytes and related markers within the gallbladder and biliary tract tissues to analyze the role of telocytes in benign biliary tract disorders.

We commence our discussion with an exploration of the mechanisms underlying the formation of cholesterol-type gallstones. Stone formation primarily arises from the excessive secretion of cholesterol within the bile. Under normal circumstances, cholesterol is absorbed by the gallbladder mucosa. However, an excess of cholesterol surpasses the gallbladder’s absorptive capacity, resulting in the accumulation of free cholesterol that can impair both mucosal and muscular functions^18^. While bile in healthy individuals may also contain saturated cholesterol, impaired gallbladder motility can lead to the visible aggregation of these microcrystals into stones. This perspective has been validated in animal models, such as the cholesterol gallstone guinea pig model, where impaired gallbladder motility was evident even before gallstone formation. In contrast, regarding the formation of bilirubin-type gallstones, Stewart^19^ proposed that bacterial infections are a pivotal factor leading to the hydrolysis of conjugated bilirubin, contributing to the formation of bilirubin-type stones. Vítek et al.^20^, on the other hand, suggested that the binding of bilirubin to free radicals might be one of the causative factors of bilirubin stones. Bilirubin radicals aggregate, deposit, and decrease the bile acid content, leading to liver cell damage and increased bilirubin concentration, thus promoting stone formation. Given the distinct etiologies of cholesterol and bilirubin gallstones, classifying them for research purposes is essential.

In summary, impaired gallbladder motility plays a pivotal role in the formation of cholesterol-type stones. Recent research in the domain of smooth muscle motility has highlighted the significance of telocytes^21^. Studies have indicated that telocytes play a role in regulating the rhythmic generation of slow-wave electrical potentials in the guinea pig gallbladder^22^. Furthermore, Balemba et al.^23^ have revealed the electrical coupling between gallbladder telocytes and smooth muscle cells in guinea pigs, and the critical role of Ca^2^ in gallbladder mitochondria in spontaneous electrical rhythm, further emphasizing the importance of telocytes in modulating gallbladder tension and motility^24^.

Telocytes play a pivotal role in tissue fibrosis, facilitating tissue repair and regeneration by establishing extensive Tps projections that connect various cells^25,26^. Research indicates that the oxygen sensitivity of telocytes may exacerbate fibrosis following myocardial infarction^27^. It is well understood that hepatic fibrosis constitutes a pathological physiological process characterized by deficient extracellular matrix (ECM) development within the liver. The secretion of ECM is predominantly orchestrated by hepatic stellate cells (HSCs), and the activation of HSCs stands as the crux of hepatic fibrosis pathology. Similarly, studies suggest that the reduction of telocytes in human liver fibrosis may influence the activation of HSCs and the deposition of ECM, potentially engaging in aberrant 3D tissue and stem cell-mediated regeneration processes^28,29^. Shah et al.^30^ also observed a diminishment of telocytes in patients with bile duct dilatation. Through comparative analysis with normal bile duct tissue, we noted varying degrees of fibrotic changes in the bile duct walls of 26 experimental cases with bile duct dilatation. Simultaneously, a conspicuous reduction of telocytes was evident in the fibrotic region of the bile duct. This reduction may result in a loss of control over the activity of fibroblasts/myofibroblasts, consequently leading to alterations in the extracellular matrix, and gradual fibrosis of the bile duct. The molecular mechanisms underlying this necessitate extensive experimental validation.

Telocytes play a pivotal role in benign biliary diseases, and the precise identification of these cells within tissue presents a key challenge in experimentation. Presently, electron microscopy remains the gold standard for discriminating telocytes. Under electron microscopy, telocytes assume an elliptical form, exhibiting elongated “telopodes” structures. These “Tps” display bead-like profiles, alternating between slender “podomers” (approximately 75–80 nm in diameter) and expanded “podoms” (approximately 250–300 nm in diameter). They house mitochondria and endoplasmic reticulum and release vesicles through their telopodes^13^. The shape of telocytes correlates with the quantity and configuration of their telopodes, forming a three-dimensional network with neighboring cells^31^. Beyond electron microscopy, the use of immunomarkers is also of paramount importance in telocyte identification. Given the heterogeneity of interstitial cells, C-kit/CD117 is considered a specific marker for telocytes within the digestive system. It is worth noting that tissue may also exhibit C-kit-positive mast cells, but mast cells possess characteristic immunomarkers such as tryptase. To obviate interference from mast cells, our experiments employ dual immunofluorescence staining using C-kit antibodies and tryptase antibodies to enhance the identification of telocytes. Therefore, our experiments consider C-kit antibody positivity and tryptase antibody negativity as the immunofluorescence markers for telocytes^17^.

In gallbladder experiments, we observed a widespread distribution of mast cells, characterized by positive Tryptase immunoreactivity, throughout various layers of the gallbladder. These cells exhibited a small, rounded morphology under immunofluorescence. In contrast, telocytes displaying positive responses to c-kit antibodies and negative responses to Tryptase antibodies were primarily situated within the muscular layer of the gallbladder. Comparative analysis of different groups revealed a marked decrease in the number of telocytes in the cholesterol-type stone group compared to the control group. Conversely, in the pigment-type stone group, telocyte counts exhibited no significant difference compared to the control group. This suggests a potential association between the reduction of telocytes and the formation of cholesterol-type stones, while the generation of pigment-type stones may not be directly linked to telocytes. This finding aligns with the results of Hu et al.^32^, who observed a decrease in the number of telocytes and a decline in gallbladder contractile function in a guinea pig model subjected to a high-cholesterol diet, as compared to the control group. The predominant distribution of telocytes near the muscular layer of the gallbladder corresponds with the findings of Pasternak et al.^33^ who identified telocytes primarily within the muscle layer, running parallel to smooth muscle cells, and classified them into two subgroups: TC-IM (intramuscular telocytes) and TC-IB (interstitial bundle telocytes). However, it is essential to note that past research has also documented telocytes distributed throughout the entire gallbladder wall, possibly due to the interference of undetected mast cells. Furthermore, in an in vitro cell culture experiment involving murine telocytes, the C-kit ligand was found to enhance the response of C-kit. Research has indicated a reduction in C-kit ligand mRNA and protein within the gallbladder of a guinea pig model subjected to a high-cholesterol diet^34^, which may be associated with sterile inflammation induced by cholesterol and disruptions in gallbladder structural function. Additionally, excessive cholesterol absorption may stimulate gallbladder cell proliferation and inflammation^35^. This corroborates our experimental results, where gallbladder mucosa significantly thickened, and muscular layer thickness decreased in the cholesterol-type stone group, as observed under HE staining. Furthermore, the cholesterol-induced cholecystic lipotoxicity^36^ was associated with defects in smooth muscle contraction and relaxation^37^, indicating a potential link between telocyte reduction and impaired gallbladder motility.

In further gallbladder motility experiments, we confirmed the positive co-expression of CCK-AR on telocytes in the human gallbladder. By adjusting the concentration of CCK-AR antibodies (within the recommended dilution range of 1:50 to 1:500), we observed that, at higher antibody concentrations, both telocytes and gallbladder smooth muscle cells exhibited positive expression. However, as the antibody concentration was reduced, gradually, only telocytes exhibited positive expression. This suggests that telocytes have a higher sensitivity to CCK compared to smooth muscle cells and may be the primary instigator of gallbladder contractions.

Reflecting on the findings of previous research teams, a reduction in CCK-AR numbers has been linked to human cholesterol gallstones. In vitro experiments have demonstrated that the addition of cholecystokinin to isolated gallbladder muscle strips results in distinct contraction frequency and amplitude. However, when telocytes were disrupted by Azure A and light exposure under the same concentration of cholecystokinin, there was a significant decrease in the contraction frequency and amplitude of the gallbladder muscle strips^38^. Our experimental results suggest that telocytes likely play a crucial role in regulating CCK-AR during gallbladder contractions. In further investigations involving Pearson correlation analyses of telocytes and CCK-AR, we successfully confirmed a positive correlation between the number of telocytes and CCK-AR cells. This validates that telocytes not only possess the CCK-AR site but are also deeply involved in regulating the functional state of gallbladder contractions. However, it is worth noting that a review of previous correlation studies has indicated that telocyte numbers are not the decisive factor influencing gallbladder contractility. This may be because these studies did not classify gallbladder stones, introducing significant bias. Furthermore, some research has found that despite the presence of gallbladder motility disorders in cholesterol gallstone patients, plasma levels of cholecystokinin remain normal or even elevated. This corroborates that gallbladder motility disorders in cholesterol gallstone patients are not due to decreased cholecystokinin concentrations. In summary, our experiments demonstrate that telocyte damage leads to a reduction in CCK-AR numbers, thereby resulting in gallbladder motility disorders.

Telocytes play a significant role in the bile duct as well. In our experiments involving the bile duct, we observed, under immunofluorescence, that Tryptase-positive mast cells exhibited a small, round morphology, distributed widely throughout the various layers of the bile duct. Simultaneously, we also noted the presence of a minority of mast cells showing double positivity for Tryptase and C-kit antibodies. Furthermore, we observed that C-kit antibody-positive, Tryptase-negative telocytes were mainly located in the muscular layer of the bile duct, aligned parallel to smooth muscle cells, with a small population of telocytes distributed in the submucosa. The majority of these telocytes exhibited a spindle-shaped morphology, although a minority displayed irregular shapes. Following immunohistochemistry of C-kit on the bile duct, we identified a noticeable reduction in C-kit-positive cells in patients with biliary dyskinesia (BD). Additionally, through further employment of double immunofluorescence staining for C-kit and Tryptase, we found that the experimental group exhibited a significantly lower number of telocytes compared to the control group. To delve deeper into the underlying causes of this phenomenon, we simultaneously conducted double immunofluorescence staining for C-kit and Caspase-3. The results revealed a higher number of Caspase-3 and C-kit double-positive cells in the experimental group compared to normal bile ducts. This suggests that increased apoptosis of telocytes may be one of the mechanisms contributing to the decreased telocyte count in the biliary dyskinesia patients’ affected bile ducts.

In this experimental segment, we aim to investigate the pathological changes in the bile duct of patients with bile duct dilatation, with a specific focus on the reduction in telocyte numbers within the experimental group. Our findings suggest that the decrease in telocyte numbers may be associated with the development of bile duct dilatation. This discovery offers a novel avenue for the histopathological study of bile duct dilatation. In summary, by examining the histological changes in bile duct dilatation, we have observed a range of histological alterations, including a decrease in telocytes, a reduction in smooth muscle cells, and an increase in bile duct fibrosis. These changes collectively contribute to the diminished tension, elasticity, and smooth muscle contractility of the bile duct wall. Furthermore, the accumulation of substances such as bile, pancreatic fluid, and stones within the bile duct gradually increases intra-biliary pressure, akin to inflating a balloon, ultimately leading to the formation of bile duct dilatation. Morphologically, this phenomenon manifests as the dilation of the bile duct.

In summarizing our experimental observations, we discern that small, round telocytes predominantly inhabit the muscular layers of both the gallbladder and the bile duct. Histological examination using Hematoxylin and Eosin (HE) staining reveals that in the gallbladders of individuals with cholesterol-type gallstones, there is an increase in mucosal thickness coupled with a reduction in muscular layer thickness when compared to the control group. Conversely, within the bile ducts of patients suffering from bile duct dilation, we observe epithelial desquamation of the mucosa and a profusion of loose, filamentous fibrous tissue, markedly deficient in muscular components. Furthermore, we observe a substantial reduction in the number of telocytes in the gallbladders of those with cholesterol-type gallstones, and this phenomenon is also manifest in regions exhibiting bile duct fibrosis. Additionally, our double immunofluorescence staining experiments targeting C-kit and CCK-AR in gallbladder tissue, combined with prior findings, lead us to speculate that telocytes potentially regulate gallbladder contractility by modulating CCK-AR. It is noteworthy that cholesterol stands as the primary causative agent in the diminution of telocytes within the gallbladder. As delineated earlier, our experiments on bile duct tissue also reveal a marked decrease in the telocyte population within the experimental group. We surmise that enhanced telocyte apoptosis induced by bile duct inflammation is one of the contributing factors to the diminished telocyte count within the pathological bile ducts of patients with bile duct dilation. In summation, we contend that telocytes play a role in the contraction of smooth muscle within both the gallbladder and the bile duct. This function may be compromised due to gallbladder inflammation resulting from cholesterol crystal formation or other causes of bile duct inflammation. It stands as a significant contributing factor to the development of benign biliary diseases. Our findings suggest a direct correlation between the reduction of telocytes in benign biliary diseases such as cholecystolithiasis and choledochal cysts, and the pathophysiology of these conditions. Although we did not conduct tests on the relationship between telocytes and biliary contraction functions, the protective role of telocytes within tissues can be reasonably inferred based on our understanding of their function in various diseases. Consequently, to deepen our understanding of the precise role stellate cells play in the biliary system, we plan to undertake comprehensive research focused on the expression and identification of stellate cells, as well as their impact on the contractile function of bile ducts.

## Data Availability

The data that support the findings of this study are available from Shenzhen People’s Hospital, China but restrictions apply to the availability of these data, which were used under license for the current study, and so are not publicly available. Data are however available from the authors upon reasonable request and with permission of Shenzhen People’s Hospital, China.
